# Independent calculation of dose from a helical TomoTherapy unit

**DOI:** 10.1120/jacmp.v10i1.2772

**Published:** 2009-02-05

**Authors:** John P. Gibbons, Koren Smith, Dennis Cheek, Isaac Rosen

**Affiliations:** ^1^ Mary Bird Perkins Cancer Center Baton Rouge Louisiana U.S.A.; ^2^ Department of Physics and Astronomy Louisiana State University and Agricultural and Mechanical College Baton Rouge Louisiana U.S.A.

**Keywords:** IMRT, TomoTherapy, monitor unit calculation, dose calculation, independent dose verification

## Abstract

A new calculation algorithm has been developed for independently verifying doses calculated by the TomoTherapy^®^ Hi·Art^®^ treatment planning system (TPS). The algorithm is designed to confi rm the dose to a point in a high dose, low dose‐gradient region. Patient data used by the algorithm include the radiological depth to the point for each projection angle and the treatment sinogram file controlling the leaf opening time for each projection. The algorithm uses common dosimetric functions [tissue phantom ratio (TPR) and output factor ($S_{\text{cp}}$)] for the central axis combined with lateral and longitudinal beam profile data to quantify the off‐axis dose dependence. Machine data for the dosimetric functions were measured on the Hi·Art machine and simulated using the TPS. Point dose calculations were made for several test phantoms and for 97 patient treatment plans using the simulated machine data. Comparisons with TPS‐predicted point doses for the phantom treatment plans demonstrated agreement within 2% for both on‐axis and off‐axis planning target volumes (PTVs). Comparisons with TPS‐predicted point doses for the patient treatment plans also showed good agreement. For calculations at sites other than lung and superficial PTVs, agreement between the calculations was within 2% for 94% of the patient calculations (64 of 68). Calculations within lung and superficial PTVs overestimated the dose by an average of 3.1% (σ=2.4%) and 3.2% (σ=2.2%), respectively. Systematic errors within lung are probably due to the weakness of the algorithm in correcting for missing tissue and/or tissue density heterogeneities. Errors encountered within superficial PTVs probably result from the algorithm overestimating the scatter dose within the patient. Our results demonstrate that for the majority of cases, the algorithm could be used without further refinement to independently verify patient treatment plans.

PACS number(s): 87.53.Bn, 87.53.Dq, 87.53.Xd

## I. INTRODUCTION

Hi·Art (TomoTherapy, Inc., Madison, WI) is a helical TomoTherapy delivery system that uses rotational intensity‐modulated radiation therapy (IMRT) to deliver optimized dose distributions.^(1)^ In the current implementation, a 64‐leaf binary multileaf collimator (MLC) is employed in a helical fashion to modulate beam delivery. This approach allows significant flexibility in the efficient delivery of intensity modulated radiation. Initial studies of this modality have demonstrated that it produces improved treatment plan dose distributions compared to conventional techniques.^(^ ^2^ ^–^ ^5^ ^)^

As with other IMRT techniques, verification of patient‐specific Hi·Art treatment plans is usually accomplished through dose measurements of the plan recomputed on a clinical measurement phantom. Such a QA procedure is valuable for testing the accuracy of the delivery system, but some errors in dose calculations will not be detected using a phantom plan evaluation.^(6)^ These include, for example, the failure to remove the planning CT couch, an incorrect patient CT dataset, or an incorrect CT‐density table. Ideally, patient‐specific verification should include both the dose calculation and dose delivery.^(6)^ Indeed, the American Association of Physicists in Medicine (AAPM) Task Group 40 recommends that the dose calculated in computer patient treatment plans be independently verified by a second check.^(7)^

We expect that the need for measurement verification of patient‐specific IMRT plans will diminish over time and be replaced by independent software checks. Many commercial IMRT delivery systems were first clinically implemented several years ago and are, therefore, well‐understood and well‐tested. In 2003, Dong et al.^(8)^ reported on the clinical validation of 751 patient cases representing nine different treatment sites. They found that the Corvus TPS was within 3.5% of point‐dose ion chamber measurements in 97% of the cases. Commercial software products are already available for verifying patient dose calculations for most IMRT delivery techniques. However, such software does not yet exist for the Hi·Art system.

Software calculation methods used for performing an independent calculation of monitor units (MUs) for other IMRT techniques^(^ ^9^ ^–^ ^20^ ^)^ are not easily applicable to Hi·Art. Methods for modeling gantry static segmental MLC (step and shoot) IMRT deliveries calculate the total dose from a modulated f eld as the sum of beamlet doses weighted by the planned leaf sequence.^(9)^ ^,^ ^(11)^ ^,^ ^(12)^ ^,^ ^(15)^ This approach was also used in the work of Ayyangar et al.^(13)^ in their calculations for dose calculations for the Peacock system. In these methods, it is assumed that the beamlet dose is not affected by the state of adjacent beamlets. However, it has been shown for Hi·Art that the dose delivered to a point under the direct path of a leaf will vary by up to 18%, depending on the state of adjacent leaves.^(21)^ ^,^ ^(22)^

Alternatively, the modulated field may be modeled as the sum of individual segments, as described by Linthout et al.^(17)^ and Chen et al.^(18)^ for gantry static dynamic MLC (sliding window) IMRT. These works use leaf sequencing files to divide the treatment into a number of individual segments. In Hi·Art, leaf sequencing is controlled by leaf sinogram files that contain the leaf opening time for each projection of the treatment delivery. These files may be used to calculate the dose from each projection, although some accounting for the modulation within a projection is required.

In this work, we introduce a technique for independently calculating dose to a point in a Hi·Art treatment plan. Our technique utilizes the planned treatment sinogram, along with dosimetry functions commonly used in standard MU calculations [tissue phantom ratio (TPR), output factor ($S_{\text{cp}}$)], obtained from gantry‐static Hi·Art beams. Calculations using this technique were compared with Hi·Art‐computed doses for a large number of phantom and patient plans. Where possible, we used dosimetry data from the TPS, rather than from measurement, in order to minimize differences introduced by measurement uncertainties and approximations in the TPS dose calculation.

## II. MATERIALS AND METHODS

### A. Point Dose Calculation Algorithm

We use the International Electrotechnical Commission (IEC) gantry coordinate system ($X_{g}$, $Y_{g}$, $Z_{g}$) for these calculations.^(23)^ The origin is taken as the intersection of the Hi·Art gantry axis with the axial CT slice containing the point of calculation. When the gantry is at 0°, the horizontal coordinate axis $X_{g}$ is directed to the viewer's right when facing the gantry from the foot of the couch, and the vertical coordinate axis $Z_{g}$ is directed upwards (towards the source) from the origin. The coordinate axis $Y_{g}$ is directed from the origin further into the Hi·Art bore. The coordinate system rotates with the gantry about $Y_{g}$ such that $Z_{g}$ is always directed toward the source.

**Figure 1:**
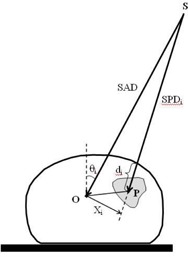
Diagram illustrating the geometry for dose calculation. For each of the 51 Hi·Art projection angles, the radiological depth $d_{i}$ is calculated along a path from the source position, *S*, to the calculation point *P*. The lateral off‐axis distance $X_{i}$ is determined by projecting this path to the plane (perpendicular to *SO*) containing the Hi·Art axis, *O. SAD* is the source‐to‐axis distance (85 cm); ${\text{SPD}}_{i}$ is the source‐to‐point distance; and $\theta_{\mathrm{03B8}}$ is the gantry angle of the projection.

The diagram in Fig. 1 illustrates the geometry for the calculation of dose $D_{p}$ to point *P*. The dose at this point is taken as the sum of the doses from each projection *i* of the Hi·Art treatment plan:

(1) $$D_{P}=\sum_{i=1}^{N_{proj}}{\dot{D}}_{P,i}\cdot t_{i}$$

where ${Ḋ}_{p,i}$ and $t_{i}$ are the dose rate and time of irradiation at point *P* for projection *i*, and $N_{\text{proj}}$ is the total number of projections.

Because the radiation field for each projection is intensity‐modulated, ${Ḋ}_{P,i}$ varies with time. To account for this, we equate the modulated projection dose to a superposition of doses from a series of constant‐intensity (i.e. unmodulated) beam segments of different field widths. The field widths of the segments are restricted to be symmetric about the ray from the source to the calculation point *P*. The time $t_{\text{ij}}$ for each segment *j* is determined as described below, such that the total dose from all segments approximates the modulated dose of projection *i*. Equation (1) may be rewritten as follows:

(2) $$D_{P}=\sum_{i=1}^{N_{proj}}\sum_{j=1}^{N_{seg,i}}{\dot{D}}_{P,ij}\cdot t_{ij},$$

where ${Ḋ}_{P,\text{ij}}$ is the dose rate to point *P* from segment *j* of projection *i*, and $N_{\text{seg},i}$ is the total number of segments within projection *i*.

**Figure 2:**
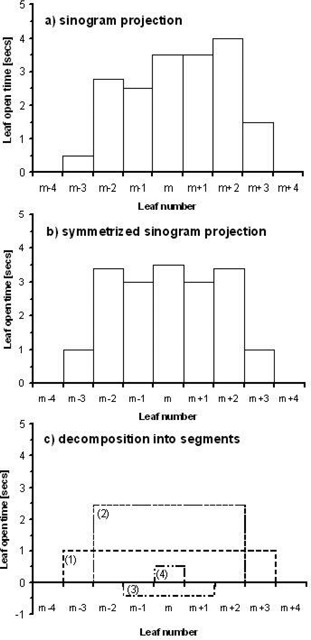
Example decomposition of a single Hi·Art projection. 2(a) Single Hi·Art sinogram projection illustrating planned leaf‐open times versus leaf number. In this example, the ray from the source to the calculation point passes through leaf number *m*. 2(b) Symmetrized sinogram projection about leaf *m* designed to deliver the same dose to the calculation point. 2(c) Decomposition of the symmetric sinogram into symmetric segments of unmodulated leaf‐open times. The summation of leaf‐open times for segments (1) to (4) results in the symmetric projection in 2(b).

The technique used to subdivide each projection is illustrated in Figs. 2(a)–2(c). Fig. 2(a) shows an example of a graph of leaf open times versus leaf position for a single Hi·Art projection.

In this case, we designated leaf number *m* as the leaf centered over the point of calculation *P*. First, the projection is approximated by an equivalent projection that is symmetric about leaf *m*. This is accomplished by setting the open times for paired‐leaves m±n to the average of their open times in the original projection. For example, leaf numbers m±3 in Fig. 2(a), which have open times of 1.5 and 0.5 seconds, respectively, are each set to an open time of 1.0 second. The resulting symmetric projection is shown in Fig. 2(b).

The projections in Figs. 2(a) and 2(b) will give the same dose to the calculation point *P* if the phantom is uniform over this region and if the energy fluence through paired leaves is equal. Although the energy fluence is not necessarily symmetric about leaf number *m*, the difference between paired leaves will be small if they are close together (i.e. if the field size is small). Additionally, the difference between the asymmetric and symmetric projections will be small when the paired‐leaf times are nearly equal, which may be the case for calculation points near the center of the planning treatment volume (PTV).

Next, the symmetric projection is decomposed into a sum of symmetric, unmodulated segments. For the example in Fig. 2, the decomposition produces four segments (Fig. 2(c). The field widths of these four segments correspond to 1, 3, 5 and 7 open MLC leaves. In general, a symmetric projection such as that in Fig. 2(b) can be written as the sum of segments with 1, 3, 5,…*n* open MLC leaves, where *n* is sufficiently large to cover the open field (i.e. n≥field width/leaf width). Thus, segment *j* corresponds to a field size containing *2j‐1* open leaves. Note that within this decomposition, it is possible to have negative segment treatment times (cf., segment 3), which, although not physically‐achievable, is acceptable for the purposes of dose calculation.

The dose from a segment *j* to point *P* is given by:

(3) $$D_{P,ij}={\dot{D}}_{0}\times {\left(\frac{85}{SPD_{i}}\right)}^{2}\times OAR_{x}(X_{i})\times \left\{t_{ij}\times S_{cp,j}\times TPR_{j}(d_{i})\times OAR_{y,j}(Y_{i},d_{i})\right\}$$

where ${Ḋ}_{0}$ is the dose rate under normalization conditions [i.e., depth $d_{0}=10\;\text{cm}$, field size $=40\times 5\;{\text{cm}}^{2}$, source‐axis distance(SAD)=85cm]. ${\text{SPD}}_{i}$ is the distance from the source to the calculation point *P* for projection *i*. $S_{\text{cp},j}$ and TPR represent the output factor and tissue phantom ratio for segment *j*, respectively. These two quantities are determined on the central axis and normalized at depth $d_{0}$. The transverse and longitudinal off‐axis ratio (OAR) functions, ${\text{OAR}}_{x}$ and ${\text{OAR}}_{y}$, account for dose rate variations with $X_{i}$ and $Y_{i}$, the lateral and longitudinal off‐axis distances of the calculation point from the central axis of projection *i*. Both OAR functions have been approximated by the dose profiles obtained from calculations of a static beam on a flat phantom. The depth‐dependent dosimetry functions (i.e. TPR, ${\text{OAR}}_{y}$) use as input the radiological depth along the projection angle in the axial plane containing the calculation point. Combining Eq. (3) with Eq. (2), the total dose at point *P* is given by:

(4) $$\begin{array}{l} D_{P}={\dot{D}}_{0}\times \sum_{i=1}^{N_{proj}}{\left(\frac{85}{SPD_{i}}\right)}^{2}\times OAR_{x}(X_{i})\\ \;\times \sum_{j=1}^{N_{seg,i}}\left\{t_{ij}\times S_{cp,j}\times TPR_{j}(d_{i})\times OAR_{y,j}(Y_{i},d_{i})\right\} \end{array}$$

If *P* is located in a high dose, low dose‐gradient region, it is assumed that the modulation within this region will be small. In this case, the majority of the dose will come from subfields with widths near the size of the PTV, reducing the relative dose contribution in Eq. (4) from small subfield sizes.

### B. Dosimetric Functions

The dosimetric data used in Eq. (4) were obtained for the 2.5‐cm and 5.0‐cm jaw settings. These data were simulated by computer and compared to measurements using a static Hi·Art field.

### B.1 Computer Simulations for Determination of Dosimetric Functions

The computer calculations of dosimetry data were made using the Hi·Art planning station to compute doses from a single projection. An artificial CT dataset was created with in‐house software to simulate a water phantom 55cm wide×40cm high×20cm long. All CT numbers inside and outside the phantom were set to create densities of 1.0 and $0.0\;g\cdot {\text{cm}}^{-3}$, respectively. The phantom was positioned within the CT image such that the anterior surface was located 10 cm above the CT axis. This arrangement was chosen to simulate a Hi·Art beam with a source‐to‐surface distance (SSD) of 75 cm and a gantry angle of 0°.

Treatment planning was started using the artificial CT dataset. The optimization process was interrupted after a few iterations and the treatment plan was archived. The binary file containing the fluence sinogram was located within the archive folder and replaced with one of several special fluence sinogram files created for this simulation. These files were created using in‐house software to produce binary sinograms corresponding to static anterior beams of varying widths. Within each special sinogram, all 64 leaves of each projection were “closed” (i.e. leaf open time=0sec), except for those in the projection at gantry angle 0° centered on the phantom. For that projection, a number of symmetric leaves were set “open” (i.e. leaves with equal, non‐zero treatment times), creating the desired field width. After replacing the file, the treatment plan was restored to the Hi·Art database. The treatment plan was restarted and the full dose was computed with the new sinogram. The final dose matrix was extracted and used to determine the parameters for Eq. (4). In our current version of the Hi·Art planning software (V. 2.2.4), this final dose matrix file (“EOPDose.img”) is available in binary format on the optimization cluster immediately after the completion of a final dose calculation. It too is accessible within a patient archive folder, also in a binary format.

The normalization dose rate $\left({Ḋ}_{0}\right)$ and dosimetric functions ($S_{\text{cp}}$, TPR, ${\text{OAR}}_{x}$ and ${\text{OAR}}_{y}$) were extracted from these static‐beam dose matrices. The dose rate for each field size on the Hi·Art axis was obtained by dividing the computed dose at 10 cm by the final sinogram time allotted to the open leaves. The dose grid point centered under a central leaf (leaf 32) was used for this calculation. Ḋ0 was set equal to the dose rate for the maximum field size $\left(40\times 5\;{\text{cm}}^{2}\right)$. $S_{\text{cp}}$ was taken as the ratio of the dose rate for a given field size to ${Ḋ}_{0}$. For use in Equation (4),$S_{\text{cp},j}$ values were tabulated for indices j=1,2,…,31 corresponding to 1, 3,…, 63 open leaves (centered about leaf number 32), and field widths of 0.625, 1.875, …, 39.375 cm at isocenter.

Percent depth doses (PDDs) were extracted from the static‐beam dose matrices and normalized at 10 cm depth. TPR values were then tabulated as above. These values were computed from the following equation:

(5) $$TPR(d,r_{d})=PDD(d,r,SSD=75)\times {\left(\frac{75+d}{85}\right)}^{2}\times \left(\frac{S_{cp}(r)}{S_{cp}(r_{d})}\right)$$

where *r* and $r_{d}$ represent the side of the equivalent square field size at the surface and 10‐cm depth, respectively. Equation (5) is an approximation to the exact relationship between TPR and PDD^(24)^ in which the ratio of total scatter factors has been used to approximate the ratio of phantom scatter factors, $S_{p}$. The difference from the exact function is expected to be small because the Hi·Art treatment head does not contain a flattening filter, thus minimizing the field size variation of collimator scatter. Additional independent calculations of TPRs using phantoms at different SSDs were made to confirm the validity of Eq. (5).

The same static‐beam dose distributions were used to determine the OARs for this calculation. The lateral profile for the maximum field width (40 cm) was used to generate the function ${\text{OAR}}_{x}$. The lateral off‐axis distances $X_{i}$ in Eq. (4) are almost always within ±15cm of the central axis. Because the lateral profile shape within the central portion of the field was not seen to vary significantly with depth, ${\text{OAR}}_{x}$ was taken as a function of off‐axis distance and jaw setting only. The lateral profile at a depth of 10 cm was used in our calculations.

The helical nature of Hi·Art beam delivery means that many projections are centered at large longitudinal distances $Y_{i}$ from the plane containing the point of calculation, where ${\text{OAR}}_{y}$ values change significantly with field size and depth. Therefore, it was necessary to tabulate ${\text{OAR}}_{y}$ as a function of both depth and field size. For each field size simulated, ${\text{OAR}}_{y}$ values were set equal to the longitudinal dose profiles taken through the center of the field. Profiles were taken for depths of 1.5, 10, 20 and 30 cm and recorded as a function of the off‐axis distance projected to the Hi·Art axis (i.e. 85 cm).

### B.2 Measurement of Dosimetric Functions

All dosimetric measurements were made using an Exradin model A1SL ion chamber (Standard Imaging, Middleton, WI) connected to a modified Keithley 602 electrometer (CNMC model K602 electrometer; CNMC Company, Nashville, TN). The ion chamber was inserted in the standard Virtual Water slab phantom provided by TomoTherapy, Inc. For all measurements, the chamber axis was aligned parallel to the $Y_{g}$‐axis (i.e. parallel to the direction of couch motion). All measurements were made in “calibrate” mode, where fixed gantry angle and couch position exposures can be made. For each measurement, integrated readings were taken for 20‐sec irradiations, with the gantry angle fixed at 0°. The accumulated charge was recorded, along with the number of MUs reported by each of the two Hi·Art monitor chambers.

$S_{\text{cp}}$ and TPR data were collected with the ion chamber placed in a Virtual Water slab phantom at the Hi·Art axis. $S_{\text{cp}}$ data were collected with the chamber centered under leaf number 32 at a depth of 10 cm. In order to ensure that the chamber was centered, measurements were made with three leaves open on the left side (i.e. leaves 29–31) and then on the right side (i.e. leaves 33–35) of leaf 32. The chamber position was adjusted laterally until the difference between these readings was minimized. Once the chamber was centered, $S_{\text{cp}}$ measurements were made using a series of specially constructed sinograms of different field sizes centered around leaf 32. $S_{\text{cp}}$ was defined by the ratio of the reading to that for the maximum field size $\left(40\times 5\;{\text{cm}}^{2}\right)$.

TPR readings were taken with the chamber positioned at depths ranging from 1.5 cm to 25 cm for both the 2.5‐cm and 5.0‐cm jaw settings. TPR is the ratio of normalized ionization readings taken at a given depth to those taken at a depth of 10 cm. Data were gathered with the central 16 and 64 MLC leaves open, corresponding to field widths of 10 cm and 40 cm at the Hi·Art axis, respectively.

### C. Dose Calculations

A dose‐calculation computer program was written to calculate Hi·Art doses using Eq. (4). The dosimetric data obtained from the planning system simulations were used in this work. This choice was made to limit the calculated dose differences to the accuracy of the algorithm alone, and exclude uncertainties introduced by measurements.

The patient‐specific information needed for calculation is the treatment planning sinogram along with the effective depth to the point of calculation for each of the 51 gantry angles used by the Hi·Art system. The treatment planning sinogram contains the leaf open times in seconds for the cumulative treatment (i.e. the total time from all fractions). It is a matrix of size $64\times N_{\text{proj}}$, where the first projection corresponds to a 0°gantry angle centered on the first slice of the planning CT dataset. The radiological depths used in the calculation were measured in the axial plane containing the point of calculation. Radiological depths were determined using our conventional treatment planning system (Pinnacle^3^; Philips Medical Systems N.A., Bothell, WA) by placing the isocenter of a conventional linac beam on the Hi·Art rotational axis. The Pinnacle^3^ planning system reports the radiological depth to any reference point that is selected to be the point of calculation. A script was written in Pinnacle^3^ to automatically compute these depths for all 51 Hi·Art gantry angles. This script exported a text file containing the depths and the calculation point coordinates. This file, along with the ASCII sinogram file, provides the inputs into the dose calculation program.

To account for the Hi·Art couch, the above mentioned Pinnacle^3^ script was run on a typical patient's pre‐treatment megavoltage CT scan from the Hi·Art machine. The script was run twice – with and without the Hi·Art couch included in the dataset. The differences in the radiological depths between these two scans were determined for all of the projections that intersected a portion of the couch. The average difference was found to be relatively small and varied little (1.2cm±0.1cm). Thus, we added 1.2 cm to the radiological depths for gantry angles that intersect the couch.

### D. Algorithm Validation

The dose calculation program was applied to a number of simple phantom geometries to confirm the validity of the algorithm. In all phantom calculations, the point used for calculating dose was located at the geometric center of the phantom. Unless stated otherwise, calculations were made using the 2.5‐cm jaw setting.

First, comparisons with Hi·Art‐calculated doses were made for treatment fields of varying lengths. A simulated CT dataset of a unit‐density cylindrical volume with diameter 20 cm and length 20 cm was created (Phantom I). Hi·Art dose distributions were computed on this phantom, which was positioned coaxial to the Hi·Art treatment axis. These computations were made with the central 16 leaves open (i.e. 10 cm width) for 1, 3 and 20 rotations about the center of the phantom, corresponding to lengths of approximately 1 cm, 3 cm, and 20 cm, respectively. The Hi·Art plans were computed with all open leaves set to the same time to eliminate the effect of modulation on the comparisons. In this case, the Hi·Art modulation factor (MF), defined as the maximum leaf open time divided by the average leaf open time for all leaves with non‐zero intensities, was set equal to one.

Second, a CT dataset of a unit‐density cylindrical volume of diameter 50 cm and length 20 cm was created to determine the depth dependence of the dose comparisons (Phantom II). This phantom was also centered so that the cylinder axis coincided with the Hi·Art axis. Two dose plans were computed with the central 16 leaves open (i.e. 10 cm width) using 4 and 29 rotations about the center of the phantom, corresponding to treatment lengths of approximately 3 cm and 20 cm. These plans were also computed with all open leaves set to the same time (i.e. MF=1).

Third, phantom calculations were made with treatment plans on a commercial cylindrical head phantom (Gammex RMI model 438 CT phantom; Gammex RMI, Middleton, WI) to determine the off‐axis and heterogeneity dependencies of the dose comparisons (Phantom III). This phantom is 20 cm long with a 20 cm diameter, and it contains heterogeneities within the phantom material. CT scans of this phantom were made with the cylindrical axis centered on (Phantom III‐A) and 10 cm off (Phantom III‐B) the center of the CT scanner. Treatment plans for these phantoms were optimized for a 7‐cm diameter PTV located at the center of each phantom using typical clinical parameters (Table I).

**Table 1:** Comparison of phantom point doses calculated with Eq. (4) and the Hi·Art Planning System.

*Phantom*	*Treatment plan*	*Modulation*	*Pitch*	*Field size* (width×length)	*Tomo plan point dose [Gy]*	*Calculated point dose [Gy]*	*Difference [%]*
				10cm×1cm (1 rotation)	60.0	59.9	−0.2%
I	Unmodulated beam for 20 cm diameter phantom	None	0.4	10cm×3cm(3rotations)	60.7	60.3	−0.6%
				10cm×20cm(20rotations)	60.5	60.0	−0.8%
II	Unmodulated beam for 50 cm diameter phantom	None	0.287	10cm×3cm(4rotations)	10.1	10.0	−0.6%
				10cm×20cm(29rotations)	10.0	9.8	−1.5%
III‐A	50 Gy to cylindrical PTV for heterogeneous	MF=1.3	0.3	7 cm PTV, on‐axis 7 cm PTV,	51.2	51.2	<0.1%
III‐B	phantom	MF=1.7		10 cm off‐axis	51.2	51.2	<0.1%

*Phantom*	*Treatment plan*	*Modulation*	*Pitch*	*Field size*	*Tomo plan point dose [Gy]*	*Calculated point dose [Gy]*	*Measured point dose [Gy]*
IV‐A	30 Gy to cylindrical PTV for	MF=1.3	0.287	5 cm PTV, on‐axis	29.9	30.0	29.8
IV‐B	TomoPhantom	MF=1.1		5 cm PTV, 11 cm off‐axis	30.5	31.0	30.6

Phantoms I and II are 20‐cm‐long cylindrical simulated water phantoms, 20 cm and 50 cm in diameter, respectively. Phantoms III‐A and III‐B are CT scans of a commercial heterogeneous cylindrical phantom (Gammex RMI model 438), 20 cm long and 20 cm in diameter. Phantoms IV‐A and IV‐B are the 30‐cm diameter Virtual Water cylindrical water phantom provided by TomoTherapy, Inc. (TomoPhantom). The axes of Phantoms I, II, III‐A, IV‐A and IV‐B were aligned coaxial with the Hi·Art axis; Phantom III‐B was scanned with the cylindrical axis offset 10 cm in the axial plane. Calculations were performed using the 2.5‐cm jaw setting for Phantoms I, II, III‐A, and III‐B, and using the 5.0‐cm jaw setting for Phantom IV‐A and IV‐B.

Finally, calculations and measurements were made for two treatment plans on the standard 30‐cm diameter cylindrical phantom available with the Hi·Art system (TomoPhantom, TomoTherapy, Inc.). A CT scan of this phantom was performed with the cylindrical axis aligned with the center of the CT scanner. The treatment plans were optimized using the 5‐cm jaw setting for a 5‐cm diameter PTV located at the center (Phantom IV‐A) and 11 cm off the center (Phantom IV‐B) of the phantom using typical clinical parameters (Table I).

Calculations were also made using the clinical treatment plans for the first 97 patients treated at our center for which sinogram files were available. For these cases, the calculation point was positioned automatically in the geometric center of the primary PTV. If this process put the point in a high dose‐gradient region or very near a tissue interface region, the point was repositioned manually. In lung cases, care was taken to place the calculation point in the target and at least 1 cm away from the low‐density lung tissue.

## III. RESULTS

### A. Dosimetric Functions

**Figure 3:**
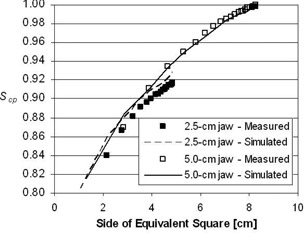
Total scatter factors S for a Hi·Art beam. Shown are measured (symbols) and simulated (lines) S values for 2.5‐cm and 5.0‐cm jaw settings. Data are determined at a depth of 10 cm and plotted versus equivalent square.

**Figure 4:**
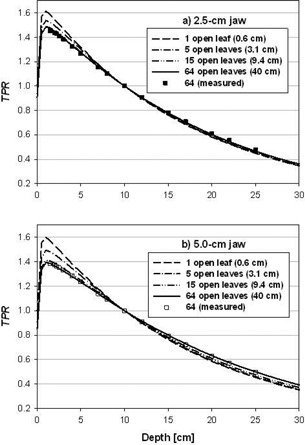
TPRs for a Hi·Art beam. Shown are measured (symbols) and simulated (lines) TPRs for jaw settings of (a) 2.5‐cm, and (b) 5.0‐cm. Data were determined in the field center and normalized to a depth of 10 cm. For each jaw setting, simulated data are shown for field widths corresponding to 1, 5, 15, and 64 open leaves, and measured data are shown for 64 open leaves.

**Figure 5:**
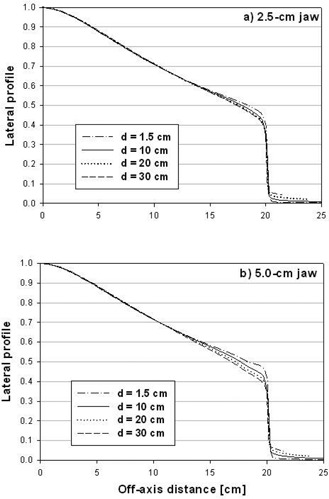
Lateral half‐profiles for a Hi·Art beam. Shown are simulated lateral profile data for jaw settings of (a) 2.5‐cm, and (b) 5.0‐cm. Data are shown for depths of 1.5, 10, 20, and 30 cm and plotted versus off‐axis position projected to 85 cm source‐to‐axis distance. The lateral profiles at a depth of 10 cm are used for the ${\text{OAR}}_{x}$ in Eq.

**Figure 6:**
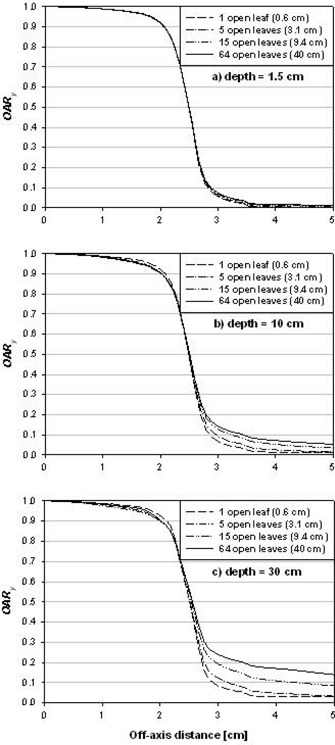
Longitudinal profiles for a Hi·Art beam. Shown are the longitudinal off‐axis ratios, ${\text{OAR}}_{y}$, computed at depths of (a) 1.5 cm, (b) 10 cm, and (c) 30 cm. Data are taken from longitudinal profiles simulated for the 5.0‐cm jaw setting. ${\text{OAR}}_{y}$ values are determined in the field center and are shown for field widths 0.6 cm, 3.1 cm, 9.4 cm, and 40.0 cm, corresponding to 1, 5, 15, and 64 open leaves, respectively.

The dosimetric data generated by computer simulation and measured with ion chamber are shown in Figs. 3 to 6. Fig. 3 shows the simulated and measured $S_{\text{cp}}$ values at 10 cm depth for the 2.5‐cm and 5.0‐cm jaw settings. The data are displayed versus the side of the equivalent square field size.^(25)^ ^,^ ^(26)^ The simulated data compare well (i.e. within 2%) with the measured $S_{\text{cp}}$ data for both jaw settings.

Figs. 4(a) and 4(b) show a sample of the simulated and measured TPR data for the 2.5‐cm and 5.0‐cm jaw settings, respectively. In general, the TPR variation with field width is significant only for field widths less than 10 cm. The TPR data for the 2.5‐cm jaw setting (Fig. 4(a) show little to no change for field widths exceeding 10 cm. The 5.0‐cm jaw data (Fig. 4(b) demonstrate a slightly greater difference with field size, although the overall variation for field widths exceeding 10 cm is still small. The simulated data compare well (i.e. within 2%) with the measured TPR data for both jaw settings.

Figs. 5(a) and 5(b) show lateral half‐profiles obtained from the Hi·Art static field simulation for the 2.5‐cm and 5.0‐cm jaw settings, respectively. Profiles at depths of 1.5, 10, 20 and 30 cm are plotted versus the off‐axis distance projected to a distance 85 cm from the source. Within the central 25 cm of the field, there is remarkably little depth dependence to the profiles for either jaw setting. For greater distances off‐axis, there is some variation, with a reduction in off‐axis dose for increasing depths. This decrease appears to be greater for the 5.0‐cm jaw setting, which is likely due to the increased contribution of scattered dose at depth for this field size.

Because of the minimal variation with depth in the central portion of the field, a single depth‐independent ${\text{OAR}}_{x}$ is used in the evaluation of Eq. (4). In this work, we have taken the lateral profile at a depth of 10 cm for this function. Although there is a variation with depth for larger off‐axis distances, for most dose calculations the lateral off‐axis distance is expected to be less than 10 cm from the Hi·Art axis. Furthermore, even for those calculation points positioned far from the axis, the projected off‐axis distance will be less than 10 cm for many, if not most, of the 51 Hi·Art gantry angles.

Figs. 6(a) to 6(c) show longitudinal off‐axis ratio, ${\text{OAR}}_{y}$, versus off‐axis distance (scaled to a distance 85 cm from the source) for depths of 1.5, 10, and 30 cm, respectively. For profiles at a depth of 1.5 cm, there is little variation in ${\text{OAR}}_{y}$ with field size. For greater depths, however, the profiles deviate from one another outside the field edge. Within the summation over projections in Eq. (4), ${\text{OAR}}_{y}$ is employed for a number of off‐axis distances inside and outside the primary field. Thus, it is critical to account for changes to ${\text{OAR}}_{y}$ both inside and outside the jaw field width.

In this work, ${\text{OAR}}_{y}$ was tabulated as a function of field size and depth. Linear interpolation with depth was made between tabulated ${\text{OAR}}_{y}$ at depths of 1.5, 10, 20, and 30 cm. Linear extrapolation for depths less than 1.5 cm or greater than 30 cm was used when necessary.

### B. Dose Calculations

The results achieved when Eq. (4) is compared to the Hi·Art‐calculated dose for a variety of phantoms are shown in Table 1. All the point doses calculated with Eq. (4) agree to within 2% with the Hi·Art calculations. Comparisons using the homogeneous 20‐cm‐diameter phantom (Phantom I) centered on the Hi·Art axis demonstrated agreement to within 1%, with a slight improvement shown in the comparisons for smaller field lengths. Comparisons for the 50‐cm‐diameter phantom (Phantom II) centered on‐axis demonstrated agreement within 2%, again with a slight improvement for the smaller field length. The calculated results agreed to within 1% of the Hi·Art‐calculated dose for the heterogeneous phantom for both the on‐axis (Phantom III‐A) and off‐axis (Phantom III‐B) plans. Finally, both the Hi·Art‐calculated doses and the doses calculated by Eq. (4) agreed within 2% of the doses measured for the 30‐cm diameter TomoPhantom (Phantom IV‐A and IV‐B).

**Figure 7:**
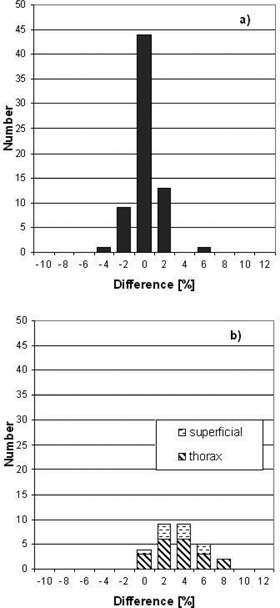
Percent difference in point dose calculations using Eq. minus that calculated by the Hi·Art planning system for 97 patient treatment plans. Shown are results for (a) 68 calculations made to regions excluding superficial and lung sites, and (b) 29 calculations made to superficial and lung sites.

Fig. 7 shows a comparison of the dose calculation with Eq. (4) with Hi·Art‐predicted point doses for 97 patient plans. The percentage differences between Eq. (4) and the Hi·Art‐calculated doses are shown in histogram form. For these plans, the target volumes ranged from 24 c^3^ to 13481 cm^3^. The modulation factors for the treatment plans ranged from 1.2 to 3.1. Fig. 7(a) shows the results for the 68 treatment plans that represent all treatment sites except those within the lung or in superficial regions. Results for these 68 sites are good, with 94% (64/68) agreeing to within 2%. The average ratio of the calculated point dose using Eq. (4) to the dose reported by the Hi·Art treatment plan is 1.004±0.013 (sample standard deviation). Fig. 7(b) shows the histogram results for the 29 treatment plans representing treatment sites within the lung or in superficial regions. As evident from Fig. 7(b), Eq. (4) systematically overestimates the doses for these sites by about 3%. Twenty of the 29 treatment plans in this group have primary PTVs located within the thorax, where the projections to the point of calculation traverse the lung. For these 29 plans, the average ratio of the calculated point dose from Eq. (4) to the dose reported by the Hi·Art treatment plan is 1.031±0.024 (sample standard deviation).

The remaining nine cases represent disease sites where the PTV was located at or near the skin surface (e.g. total scalp, chest wall). In each of these cases, the point of calculation was placed within 3 cm of the surface, with a minimum depth of 1 cm in all cases. The results for these calculations appear similar to those for lung; the algorithm overestimates the dose compared to the calculations from the Hi·Art planning system. For these nine plans, the average ratio of the calculated point dose from Eq. (4) to the dose reported by the Hi·Art treatment plan is 1.032±0.022 (sample standard deviation).

## IV. DISCUSSION

The agreement between the simulated and measured static field dosimetric data was good. This is expected, assuming the beam modeling process during Hi·Art commissioning is accurate in reproducing simple static‐field doses. Because the intent of this work was to investigate the accuracy of the algorithm in verifying point doses, the simulated dosimetry data were used to remove measurement uncertainties from the comparisons.

It was determined through this investigation that the dosimetric function that most significantly affected the results was the longitudinal off‐axis ratio. We conducted a number of trials using simpler approximations to ${\text{OAR}}_{y}$ prior to implementing the current methodology. These trials included a simple pillbox function (${\text{OAR}}_{y}=1$ within the field; 0 or some small fixed value outside) and a depth‐independent ${\text{OAR}}_{y}$ equal to longitudinal profiles at some fixed depth or field size. However, the data in Figs. 6(a) to 6(c) demonstrate that the variation with both field size and depth are sufficiently large to require a corresponding dependency in ${\text{OAR}}_{y}$. The requirement is greater for larger field lengths, where the tails of the longitudinal profile comprise a greater portion of the calculation summation.

The agreement between the point doses calculated with Eq. (4) and the Hi·Art‐calculated doses is within 2% for all phantoms studied in this work. Each of these phantom plans was designed to test a particular portion of the algorithm, including calculations at different depths, off‐axis positions, and field lengths. The increasing error with field length for phantoms I and II may reflect the sensitivity of the calculation to changes in ${\text{OAR}}_{y}$ with depth at large off‐axis distances.

As shown in Fig. 7(a), comparisons with patient plans excluding lung and superficial regions are in good agreement. The majority of these treatment plans involved prostate or head and neck diseases, although other plans within the pelvis, abdomen, and central nervous system were included. Such close agreement is surprising given both the complex nature of the Hi·Art treatment and exclusion of longitudinal off‐axis changes in the patient anatomy in Eq. (4).

For the lung plans (Fig. 7(b), the average difference is about 3%, but differences as high as 8% were found in this study. By using the radiological depth for the calculations in Eq. (4), the algorithm effectively applies a ratio of TPR correction factor to a homogeneous calculation. This correction factor is relatively easy to incorporate without requiring knowledge of the location of the calculation point with respect to tissue heterogeneities. The overestimate with this type of calculation in regions just beyond a low‐density heterogeneity is consistent with the results of Mackie et al.,^(27)^ who measured doses in and beyond lung phantom material. Better agreement within these regions might be obtained if a more complicated algorithm were used (e.g. Batho), although additional information would have to be extracted from the treatment planning system. This shortfall is not unique to Hi·Art calculations; any MU‐check algorithm that uses radiological depth will suffer from the same deficiencies.

For the superficial plans compared in Fig. 7(b), the average difference is also about 3%, with a maximum difference of about 7%. In all of these cases, the PTV was located at or near the tissue surface, with a 0.5‐cm to 1.0‐cm layer of bolus material included in the CT dataset. In order to minimize the dose to the underlying normal tissues, it is expected that the majority of the dose delivered by the Hi·Art beam would come from tangential or near‐tangential beams. It is not surprising, therefore, to have larger discrepancies when using data gathered from fl at‐phantom and normal incidence geometries. Furthermore, the reduction in scatter from that portion of the beam at or outside the skin surface is not accounted for within the algorithm. This missing scatter may explain the overestimate of dose in the current approach.

Regardless of the larger difference found in these latter cases, the current algorithm is suitable for a second check of the Hi·Art‐calculated dose. It is used for all patients in our clinic. The second check process is reasonably efficient. The total time required to perform the dose calculation, including obtaining the radiological depths and treatment sinogram, was less than ten minutes. At a minimum, large Hi·Art treatment planning errors should easily be detected for all cases, whereas smaller errors may be detectable for some treatment sites.

## V. CONCLUSIONS

A new calculation algorithm that uses standard dosimetric functions has been developed for use in verifying point doses from the Hi·Art treatment planning system. It was tested using dosimetry data extracted from the TPS by simulation of static, unmodulated fields. When compared to Hi·Art plans for a variety of cylindrical phantom geometries and treatment deliveries, the calculation agreed to within 2%. When tested on patient cases with a variety of target sites excluding lung and superficial targets, the average ratio of the calculation to the Hi·Art plan was 1.004±0.013 (sample standard deviation) and 94% of the sample cases agreed to within ±2%. For patients with lung and superficial targets, the calculation was systematically higher than the Hi·Art calculation, resulting in an average ratio of 1.031±0.024 (sample standard deviation).

For lung targets, improvement in the algorithm could be achieved by implementing a more accurate heterogeneity correction than the radiological path length method currently used. For superficial targets, the deficit in lateral scatter for tangential beams might explain the overestimate of the current approach. To account for this effect, additional patient‐specific information would be required (e.g. knowledge of the patient's external contour on the axial slice containing the point of calculation). Nevertheless, in its present form this new calculation algorithm has demonstrated an accuracy with 2% for the majority of patient treatment plans.

The clinical implementation of this methodology would require appropriate machine and patient‐specific data. To be effective as an independent second check, it should include measured dosimetry data and patient depths obtained from a separate software system. Commercial software tools for easily obtaining patient radiological depths and Hi·Art sinograms would make this method an attractive supplement or alternative to patient‐specific measurements in most cases.

## ACKNOWLEDGEMENTS

This work was supported in part by a research agreement from TomoTherapy, Inc.

## References

[1] Mackie TR . History of tomotherapy. Phys Med Biol. 2006;51(13):R427–53. 16790916 10.1088/0031-9155/51/13/R24

[2] Orton N , Jaradat H , Welsh J , Tome W . Total scalp irradiation using helical tomotherapy. Med Dosim. 2005;30(3):162–68. 16112468 10.1016/j.meddos.2005.05.002

[3] Han C , Liu A , Schultheiss TE , Pezner RD , Chen YJ , Wong JY . Dosimetric comparisons of helical tomotherapy treatment plans and step‐and‐shoot intensity‐modulated radiosurgery treatment plans in intracranial stereotactic radiosurgery. Int J Radiat Oncol Biol Phys. 2006;65(2):608–16. 16690442 10.1016/j.ijrobp.2006.01.045

[4] Fiorino C , Dell'Oca I , Pierelli A , et al. Significant improvement in normal tissue sparing and target coverage for head and neck cancer by means of helical tomotherapy. Radiother Oncol. 2006;78(3):276–82. 16546279 10.1016/j.radonc.2006.02.009

[5] Hui SK , Kapatoes J , Fowler J , et al. Feasibility study of helical tomotherapy for total body or total marrow irradiation. Med Phys. 2005;32(10):3214–24. 16279075 10.1118/1.2044428

[6] Ezzell GA , Galvin JM , Low D , et al. Guidance document on delivery, treatment planning, and clinical implementation of IMRT: report of the IMRT Subcommittee of the AAPM Radiation Therapy Committee. Med Phys. 2003;30(8):2089–115. 12945975 10.1118/1.1591194

[7] Kutcher GJ , Coia L , Gillin M , et al. Comprehensive QA for radiation oncology: report of AAPM Radiation Therapy Committee Task Group 40. Med Phys. 1994;21(4):581–618. 8058027 10.1118/1.597316

[8] Dong L , Antolak J , Salehpour M , et al. Patient‐specific point dose measurement for IMRT monitor unit verification. Int J Radiat Oncol Biol Phys. 2003;56(3):867–77. 12788197 10.1016/s0360-3016(03)00197-4

[9] Boyer A , Xing L , Ma CM , et al. Theoretical considerations of monitor unit calculations for intensity modulated beam treatment planning. Med Phys. 1999;26(2):187–95. 10076972 10.1118/1.598502

[10] Ma CM , Pawlicki T , Jiang SB , et al. Monte Carlo verification of IMRT dose distributions from a commercial treatment planning optimization system. Phys Med Biol. 2000;45(9):2483–95. 11008950 10.1088/0031-9155/45/9/303

[11] Kung JH , Chen GT , Kuchnir FK . A monitor unit verification calculation in intensity modulated radiotherapy as a dosimetry quality assurance. Med Phys. 2000;27(10):2226–30. 11099189 10.1118/1.1286553

[12] Xing L , Chen Y , Luxton G , Li JG , Boyer AL . Monitor unit calculation for an intensity modulated photon field by a simple scatter‐summation algorithm. Phys Med Biol. 2000;45(3):N1–7. 10730973 10.1088/0031-9155/45/3/401

[13] Ayyangar KM , Saw CB , Shen B , Enke CA , Nizin PS . Independent dose calculations for the PEACOCK System. Med Dosim. 2001;26(1):29–35. 11417504 10.1016/s0958-3947(00)00057-1

[14] Watanabe Y . Point dose calculations using an analytical pencil beam kernel for IMRT plan checking. Phys Med Biol. 2001;46(4):1031–38. 11324949 10.1088/0031-9155/46/4/309

[15] Yang Y , Xing L , Li JG , et al. Independent dosimetric calculation with inclusion of head scatter and MLC transmission for IMRT. Med Phys. 2003;30(11):2937–47. 14655941 10.1118/1.1617391

[16] Ma CM , Price RA, Jr. , Li JS , et al. Monitor unit calculation for Monte Carlo treatment planning. Phys Med Biol. 2004;49(9):1671–87. 15152923 10.1088/0031-9155/49/9/006

[17] Linthout N , Verellen D , Van Acker S , Storme G . A simple theoretical verification of monitor unit calculation for intensity modulated beams using dynamic mini‐multileaf collimation. Radiother Oncol. 2004;71(2):235–41. 15110458 10.1016/j.radonc.2004.02.014

[18] Chen X , Yue NJ , Chen W , et al. A dose verification method using a monitor unit matrix for dynamic IMRT on Varian linear accelerators. Phys Med Biol. 2005;50(23):5641–52. 16306658 10.1088/0031-9155/50/23/016

[19] Fan J , Li J , Chen L et al. A practical Monte Carlo MU verification tool for IMRT quality assurance. Phys Med Biol 2006; 51 (10):2503–15. 16675866 10.1088/0031-9155/51/10/010

[20] Baker CR , Clements R , Gately A , Budgell GJ . A separated primary and scatter model for independent dose calculation of intensity modulated radiotherapy. Radiother Oncol. 2006;80(3):385–90. 16956682 10.1016/j.radonc.2006.08.011

[21] Balog JP , Mackie TR , Reckwerdt P , Glass M , Angelos L . Characterization of the output for helical delivery of intensity modulated slit beams. Med Phys. 1999;26(1):55–64. 9949398 10.1118/1.598477

[22] Balog J , Olivera G , Kapatoes J . Clinical helical tomotherapy commissioning dosimetry. Med Phys. 2003;30(12):3097–106. 14713076 10.1118/1.1625444

[23] International Electrotechnical Commission . Radiotherapy equipment‐Coordinates, movements and scales. Amendment to Report No. 61217 (2002). IEC. 2007.

[24] Khan FM . The Physics of Radiation Therapy. 3rd. rev. ed. Philadelphia: Lippincott Williams & Wilkins; 2003 560 p.

[25] Day MJ , Aird EG . The equivalent field method for dose determinations in rectangular fields. BJR Suppl 1996;25:138–51. 9068361

[26] Clarkson J . A note on depth doses in fields of irregular shape. British Journal of Radiology. 1941;14:265–68.

[27] Mackie TR , el‐Khatib E , Battista J , Scrimger J , Van Dyk J , Cunningham JR . Lung dose corrections for 6‐ and 15‐MV x rays. Med Phys. 1985;12(3):327–32. 3925308 10.1118/1.595691

